# Post-Intensive Care Syndrome in Non-COVID-19 ICU Survivors during the COVID-19 Pandemic in South Korea: A Multicenter Prospective Cohort Study

**DOI:** 10.3390/jcm11226653

**Published:** 2022-11-09

**Authors:** Jiyeon Kang, Jiwon Hong, Jin-Heon Jeong

**Affiliations:** 1 College of Nursing, Dong-A University, Busan 49201, Korea; 2 Department of Nursing, Youngsan University, Yangsan-si 50510, Korea; 3 Department of Intensive Care Medicine & Neurology, Dong-A University Hospital, College of Medicine, Dong-A University, Busan 49201, Korea

**Keywords:** COVID-19, post-intensive care syndrome, South Korea, prospective cohort study

## Abstract

A prospective observational cohort study investigated the prevalence of post-intensive care syndrome (PICS) among non-COVID-19 ICU survivors during the COVID-19 pandemic. Adults who had been admitted to the ICU for more than 24 h were enrolled, and followed-up at 3, 6, and 12 months post-discharge. PICS (mental health, cognitive, and physical domains) was measured using the Hospital Anxiety and Depression Scale, Posttraumatic Diagnosis Scale, Montreal Cognitive Assessment, and Korean Activities of Daily Living (ADL) scale. Data were analyzed from 237 participants who completed all three follow-up surveys. The prevalence of PICS was 44.7%, 38.4%, and 47.3%, at 3, 6, and 12 months of discharge, respectively. The prevalence of PICS in the mental health and cognitive domains decreased at 6 and increased at 12 months. The prevalence of PICS in the physical domain declined over time. Changes in PICS scores other than ADL differed significantly according to whether participants completed follow-up before or after December 2020, when COVID-19 rapidly spread in South Korea. In the recent group, anxiety, depression, post-traumatic stress disorder, and cognition scores were significantly worse at 12 months than at 6 months post-discharge. The COVID-19 pandemic may have adversely affected the recovery of non-COVID-19 ICU survivors.

## 1. Introduction

Declining intensive care unit (ICU) mortality rates have drawn attention to the recovery and long-term outcomes of critical care survivors. ICU patients go through a recovery process for a certain period after discharge, regardless of their type of critical illness. Recent longitudinal studies have reported that survivors suffer from various impairments for at least 1–5 years after ICU discharge [1]. Newly developed or worsened mental health and cognitive and physical impairments associated with critical care are referred to as post-intensive care syndrome (PICS). PICS not only negatively affects the health-related quality of life of survivors but also burdens their families and society [2,3].

Determining the trajectory of post-ICU recovery can help determine when to provide and evaluate necessary interventions. Several theories explaining the recovery trajectory from illness suggest that major recovery from critical illness occurs within 12 months [4]. Prospective follow-up studies of ICU survivors report that functional decline at 3–6 months after discharge tends to improve approximately 12 months after discharge. In a longitudinal study of 406 ICU survivors, the prevalence of PICS decreased from 64% at 3 months to 56% at 12 months after discharge, and the prevalence of PICS at 12 months was lower in all three domains [5]. A tendency toward gradual recovery within 12 months has also been observed in survivors’ cognitive function [6], physical function [7], and quality of life [8].

The recent COVID-19 pandemic poses a major threat to society, especially to the healthcare sector. The mortality rate in critical care patients with COVID-19-related conditions has reached 33% [9], and one study found that 91% of COVID-19 ICU patients experience PICS after discharge [10]. Threats to critically ill patients include delays in recovery from non-COVID-19-related illness, as well as hospital-acquired COVID-19 and death. Isolation from family and increased delirium owing to the policy of limiting ICU visits during the pandemic may increase the risk of PICS in critical care survivors with non-COVID-19-related conditions [11].

There is a lack of information on PICS and recovery after discharge in the general ICU population during the COVID-19 pandemic. This study investigated the prevalence of PICS among ICU survivors from the beginning of the COVID-19 pandemic. The primary research objective was to determine the prevalence of PICS in ICU survivors at 3, 6, and 12 months after discharge. A secondary objective was to determine whether there were any differences in mental health, cognitive, and physical impairments based on the duration post-discharge.

## 2. Materials and Methods

### 2.1. Design

This was a prospective observational cohort study, conducted in 19 ICUs of four university hospitals in Busan, South Korea between 11 June 2019 and 27 July 2021. The study protocol was registered with the Korean Clinical Research Information Service (No. #KCT0004045).

### 2.2. Participants

**Figure 1:**
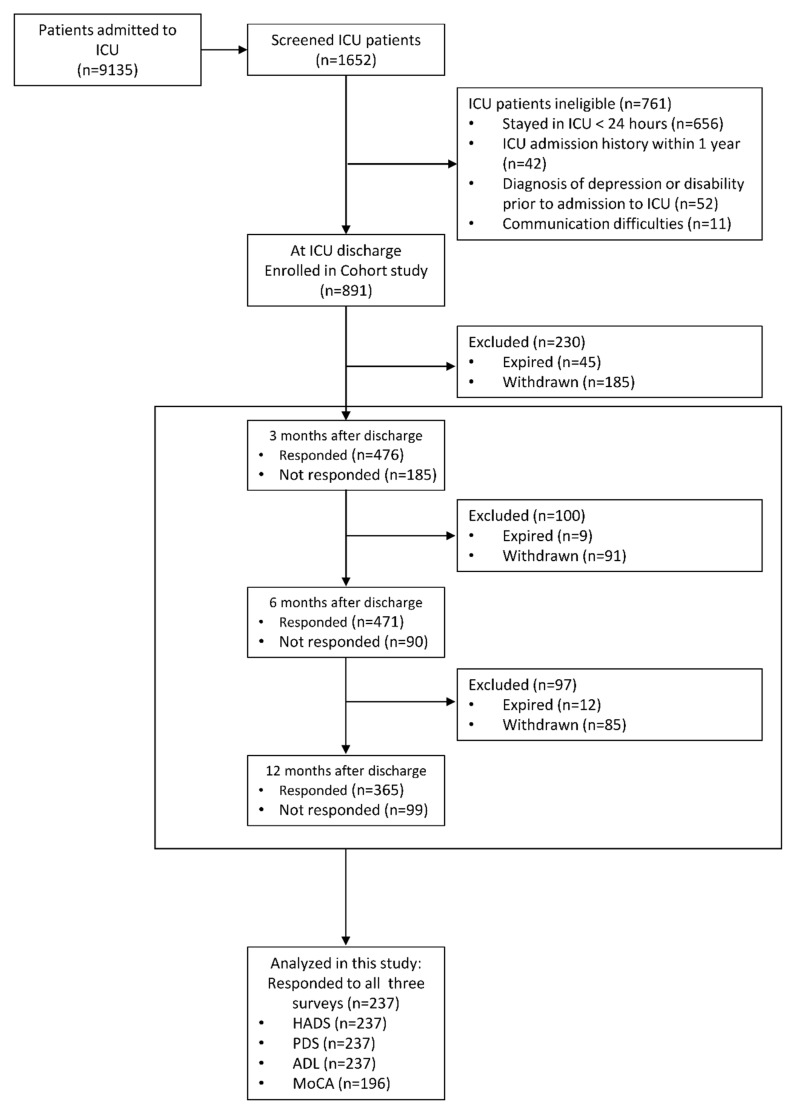
Flow diagram of study participants. Abbreviations: ADL: Activities of Daily Living; HADS: Hospital Anxiety and Depression Scale; ICU: intensive care unit; MoCA: Montreal Cognitive Assessment; PDS: Post-traumatic Diagnosis Scale.

A total of 9135 patients were admitted to the participating ICUs during the enrollment period. Among them, 891 adults aged 18 years or older who were admitted to the ICU for more than 24 h were enrolled. Exclusion criteria were those who had been previously admitted to an ICU within the previous year, those with mental health and physical disabilities before admission, and those who had difficulty responding to the survey questionnaire owing to limited communication ability. None of the participants had critical illness related to COVID-19. We analyzed the data of 237 survivors who participated in all three follow-up surveys conducted 3, 6, and 12 months after discharge. As 41 of the 237 participants missed at least one follow-up cognitive assessment, we analyzed the data of the mental health and physical domains of 237 participants and the cognitive domain of 196 participants (Figure 1).

### 2.3. Measures

We measured PICS using established tools to evaluate symptoms affecting the three core domains of PICS. At the beginning of the follow-up, the participants responded to the questionnaire either face-to-face or by telephone. As the COVID-19 situation worsened, all follow-up questionnaire surveys were eventually conducted by telephone. Five trained research assistants collected the data. They attended two standardization workshops for surveyors, including a Montreal Cognitive Assessment (MoCA) application training. PICS was defined as an impairment in any one of the mental health, cognitive, or physical domains. The measurement tools and cut-off points for the three PICS domains were as follows.

#### 2.3.1. Mental Health

We measured anxiety, depression, and post-traumatic stress disorder (PTSD) as the mental health domain of PICS.

Anxiety and depression were assessed using the Hospital Anxiety and Depression Scale (HADS) developed by Zigmond and Snaith [12]. The HADS comprises 14 items: 7 anxiety items (HADS-A) and 7 depression items (HADS-D). It uses a four-point Likert scale, with possible scores ranging from 0 to 21 for both anxiety and depression. Higher scores indicate higher levels of anxiety and depression. A total score of 0–7, 8–10, and ≥11 is classified as normal, mild anxiety or depression, and severe anxiety or depression, respectively [12]. In this study, anxiety and depression were defined as a HADS-A or HADS-D score of ≥8.

PTSD symptoms were assessed using the Korean version of the Post-traumatic Diagnosis Scale (PDS) [13,14]. The PDS has 17 items that are measured using a four-point Likert scale. The score ranges from 0–51; a total score of 1–10, 11–20, 21–35, and ≥36 is considered as mild, moderate, moderate-to-severe, and severe symptoms, respectively [14,15]. In this study, PTSD was defined as a PDS score of ≥11.

#### 2.3.2. Cognitive Impairment

The cognitive domain of PICS was measured using MoCA for face-to-face interviews and MoCA-BLIND for telephone interviews [16,17]. MoCA comprises 32 items that assess global cognition, including attention, concentration, executive function, memory, language, visuospatial skills, abstraction, calculation, and orientation. MoCA-BLIND contains the same items as the original MoCA, excluding those requiring visual ability. The score ranges from 0–30 for MoCA and 0–22 for MoCA-BLIND. We converted the MoCA-BLIND score into a MoCA score (https://www.mocatest.org/faq/, accessed on 7 November 2022), and a total score of <23, the reference point for mild cognitive impairment [17,18,19], was defined as cognitive impairment.

#### 2.3.3. Physical Impairment

The physical domain of the PICS was measured using the Korean Activities of Daily Living (K-ADL) scale [20]. The K-ADL consists of seven items on activities, such as dressing, grooming, bathing, feeding, mobility, toilet use, and bowel and bladder control. The K-ADL uses a three-point Likert scale, with a higher score indicating greater dependence in daily activities. In this study, physical impairment was defined as a total K-ADL score of ≥8 [20].

### 2.4. Statistical Analysis

The demographic and ICU treatment-related characteristics of the participants were reported using descriptive statistics, including means and standard deviations. The prevalence of PICS and of each PICS domain was calculated as the proportion (%) of participants who satisfied the definition. Differences in PICS score according to the time were analyzed using one-group repeated-measures ANOVA.

We divided the participants into two groups to further analyze the change in the PICS score according to the period. Group A comprised participants who completed all follow-ups prior to December 2020, when COVID-19 began to spread rapidly in South Korea [21]. Group B comprised participants who completed follow-up between December 2020 and July 2021. Differences in demographic and treatment-related characteristics between the two groups were analyzed using the *t*-test or χ^2^ test for continuous and categorical measures, respectively. Repeated-measures ANOVA was used to analyze whether there was a difference in the PICS score change according to the calendar period between groups A and B. Statistical significance was defined as α < 0.05. All of the data analyses were conducted in SPSS version 27 (IBM Corporation, Armonk, NY, USA).

## 3. Results

### 3.1. Participant Characteristics

The demographic and ICU-treatment-related characteristics of the 891 participants who enrolled in the cohort study immediately after discharge from the ICU are shown in Supplementary Table S1. The mean age of the 237 participants who participated in all three surveys at 3, 6, and 12 months after discharge was 58.3 ± 13.3 years, and 60.3% were men. More than half were employed and had one or more functional comorbidities prior to ICU admission. During the ICU admission, 16.0% of the participants received mechanical ventilator treatment and 10.5% experienced delirium. The mean Simplified Acute Physiology Score was 31.92 ± 15.08 and the mean Acute Physiology and Chronic Health Evaluation II score was 10.70 ± 5.81. Participants stayed in the ICU for a mean of 4.02 ± 4.28 days, and most were discharged home (Table 1).

### 3.2. Prevalence of PICS

**Figure 2:**
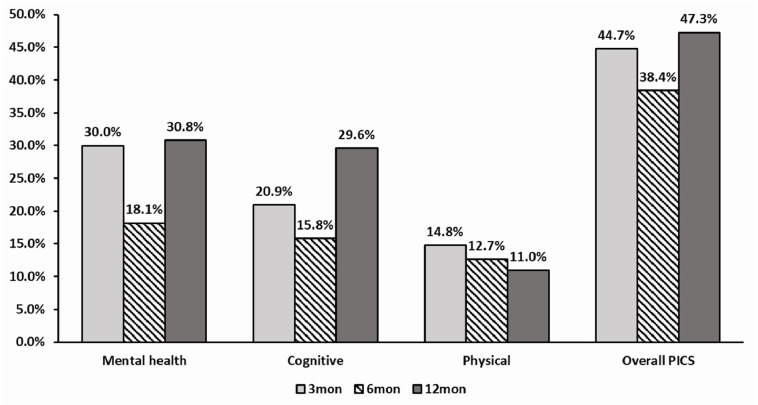
Prevalence of post-intensive care syndrome at 3, 6, and 12 months after discharge.

The overall PICS prevalence was 44.7%, 38.4%, and 47.3% at 3, 6, and 12 months after discharge, respectively. The prevalence of PICS at 3, 6, and 12 months were 30.0%, 18.1%, and 30.8%, respectively, in the mental health domain; 20.9%, 15.8%, and 29.6%, respectively, in the cognitive domain; and 14.8%, 12.7%, and 11.0%, respectively, in the physical domain (Figure 2, Supplementary Table S2). The anxiety, depression, PTSD, cognition, and ADL scores were better at 6 months than at 3 months but worsened again at 12 months, and these non-linear changes over time were statistically significant (Table 2).

### 3.3. Effect of Calendar Period on the Prevalence of PICS

**Figure 3:**
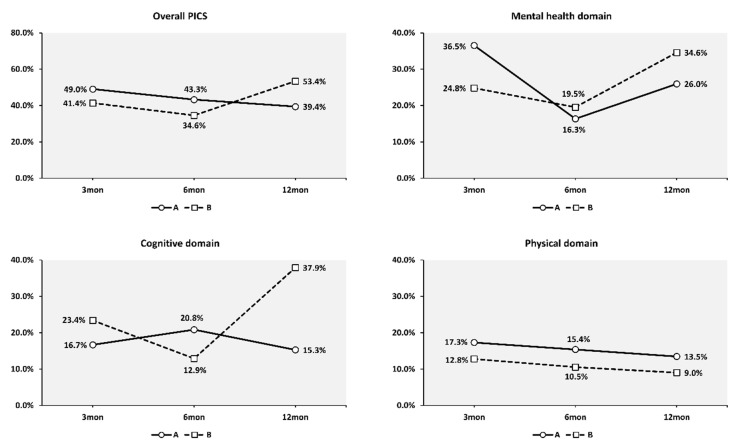
Post-intensive care syndrome prevalence in Groups A and B.

The overall PICS prevalence among participants in Group A, who completed the follow-up before the rapid spread of COVID-19 in South Korea, was 49.0%, 43.3%, and 39.4% at 3, 6, and 12 months after discharge, respectively. However, unlike participants in Group A, where the prevalence of PICS decreased over time, the prevalence of PICS among participants in Group B decreased from 41.4% at 3 months to 34.6% at 6 months, and then increased to 53.4% at 12 months. The prevalence of PICS by domain in the two groups is shown in Figure 3 and Supplementary Table S2. The repeated-measures ANOVA of the 3-, 6-, and 12-month scores of the PICS variables revealed significant time (since discharge) and group (calendar period) interactions in the anxiety, depression, PTSD, and cognition scores. However, there was no significant difference in the trends in the ADL scores over time between the two groups (Table 3).

## 4. Discussion

This study investigated the prevalence of PICS among ICU survivors during the COVID-19 pandemic. As no participants had COVID-19, we observed changes in PICS in general ICU patients during the COVID-19 pandemic. In the 237 participants who were admitted to the ICU for more than 24 h and completed follow-up assessments at 3, 6, and 12 months after discharge, almost half experienced PICS within 12 months of discharge. Changes in the physical domains of the study participants were consistent with the usual recovery trajectories from critical illness or other chronic diseases [4]. However, in the mental health and cognitive domains, there was a trend toward an initial improvement between 3 and 6 months, followed by a worsening between 6 and 12 months. These changes in both domains were more pronounced in Group B (vs. Group A) participants, who completed the follow-up survey after the surge in COVID-19 cases in December 2020 and are therefore likely to have been more affected by the pandemic during their recovery period.

Survivors of critical illness have persistent impairment of physical function, which recovers over months or years. Mental and cognitive impairment after ICU admission also gradually improved [5]. In patients admitted to the ICU for sepsis, depressive symptoms resolved in 88% of patients during a 1-year follow-up period [22]. In ICU survivors with shock or respiratory failure, cognitive impairment decreased from 47% at 3 months to 42% at 12 months [6]. In contrast, the overall PICS prevalence in our study decreased between 3 and 6 months and then increased between 6 and 12 months. This trend became more pronounced after the surge in the incidence of COVID-19 in South Korea in December 2020. This suggests that the COVID-19 pandemic may have impeded recovery and exacerbated the effects of PICS on mental health and cognition.

Although survivors’ disease and pre-hospital conditions, and other factors can influence the trajectory of recovery from critical illness, recovery tends to be apparent early on after ICU discharge and more slowly over time [4,23,24]. The impact of COVID-19 pandemic on the recovery of survivors in our cohort was not evident in the early stages of rapid recovery but became evident at 12 months. Our results contribute to understanding the recovery trajectory from critical illness and guide the timing of rehabilitative interventions for PICS.

The impact of the COVID-19 pandemic on PICS was more evident when the PICS recovery trends of the two groups were compared. In Group A, the overall PICS prevalence decreased over the 12-month follow-up period, whereas in Group B, which was followed-up during the period after COVID-19 surged, the prevalence of PICS increased by almost 20% at 12 months. When compared by each domain of PICS, the prevalence of PICS in the physical domain decreased over 12 months in both groups, suggesting that physical recovery was not affected by the COVID-19 surge. In the mental health domain, the prevalence of PICS was lower at 6 months than at 3 months, and higher at 12 months than at 6 months in both groups. Group B had less recovery at 6 months and a more marked increase at 12 months, suggesting that the COVID-19 surge had a negative impact on the recovery of mental health. Both groups showed opposite recovery progress in the cognitive domain of PICS. The prevalence of PICS in the cognitive domain at 12 months differed by more than 20%, suggesting that the COVID-19 surge might have had an adverse effect on cognitive function.

The COVID-19 pandemic has had a negative effect on the mental health of critically ill patients. In COVID-19 patients who have survived ICU admission, psychological distress is common during the first 6 months after hospital discharge [25]. The COVID-19 pandemic has driven people into social isolation and has had a negative impact on the mental health of the general population. A cross-sectional study showed that anxiety, stress, and depression have been prevalent worldwide during the COVID-19 pandemic [26]. In addition, the COVID-19 pandemic has been associated with an increased incidence of PTSD, in addition to an increased prevalence of mental health problems such as depression and anxiety [27]. In our study, Group B appeared to be more affected by the pandemic during their recovery from PICS than Group A. During the follow-up period of Group B, the number of confirmed COVID-19 cases in South Korea surged [21], and accumulated psychological fatigue may have increased as quarantine guidelines, such as social distancing, were constantly extended and strengthened.

Our findings suggest that the COVID-19 pandemic may have negatively impacted the recovery of cognitive function in ICU survivors. The communication and mood of adults with mild cognitive impairment worsened during the pandemic [28]. Emotional responses, such as anxiety and depression, related to the COVID-19 pandemic were significantly associated with negative cognition, including paranoia and compulsion [29]. This indirect effect of the pandemic on cognition can be explained by a decrease in interpersonal relationships and interactions owing to quarantine and social distancing. Reduced interactions with family, relatives, and friends can negatively affect cognitive function and quality of life [30]. Additionally, psychological and physical distress caused by the pandemic has increased the burden on caregivers, and this may have had a negative effect on cognition by undermining patients’ sense of security because of changes in their regular daily routine [28].

PICS affects the recovery process, reduces quality of life, and burdens the healthcare system. PICS, especially physical and cognitive impairment, is also associated with later mortality in ICU survivors [31]. Nonpharmacologic interventions, including exercise and ICU diaries, can mitigate the adverse outcomes of PICS [32]. However, social isolation during the COVID-19 pandemic has limited these face-to-face interventions. Recent studies have reported that non-face-to-face interventions using online platforms or smartphone applications are effective in maintaining and improving mental health [33,34] and preventing the onset of disability [33]. Hence, it is necessary to develop and implement non-face-to-face interventions for the prevention of PICS in ICU survivors. Understanding the recovery process from PICS and applying appropriate interventions are important for improving long-term outcomes in ICU survivors.

This study was conducted prospectively, and all tertiary care hospitals in Busan city participated. More than 350,000 patients are admitted to ICUs in South Korea every year [21], and this number has been increasing [35]. Few studies have evaluated changes in PICS over the course of the recovery period. Additionally, although the number of studies reporting the results of PICS-related prospective studies is increasing, most studies do not report all three domains of PICS [23]. To our knowledge, no prospective studies to date have comprehensively reported on the mental health, cognitive, and physical impairments experienced by ICU survivors in South Korea. We investigated all three domains of PICS over a 1-year-follow-up period.

Our study has some limitations. First, not all ICU survivors participated; thus, there is a possibility of selection bias. Second, only 237 of the 891 participants who enrolled completed all three follow-ups and were included in the analysis. Participants who completed the 1-year-follow-up tended to be young, with less severe disease, and had shorter ICU stays (Supplementary Table S1). Among the survivors, it is possible that patients with less severe PICS were more likely to continue participating. This may limit the generalizability of the results. Third, although there was a significant difference in the occurrence of PICS before and after the COVID-19 surge, the results alone cannot confirm a relationship between the COVID-19 pandemic and the increased prevalence of PICS during the latter part of the study period. Further studies are required to determine the relationship between the COVID-19 pandemic and PICS.

## 5. Conclusions

Almost half of the study participants experienced PICS during the COVID-19 pandemic, and the prevalence increased during the study period especially at the 12-month follow-up post-discharge. This suggests that the COVID-19 pandemic may have affected recovery from PICS, particularly in the mental health and cognitive domains. With no end to the COVID-19 pandemic in sight, non-face-to-face interventions are needed to help ICU survivors recover successfully.

## Figures and Tables

**Table 1:** Baseline characteristics.

Variables	Categories	Total (N = 237)	Group A (N = 104)	Group B (N = 133)	*p*
Age, years		58.30 ± 13.30	57.55 ± 12.73	58.89 ± 13.75	0.443
Male sex		143 (60.3)	64 (61.5)	79 (59.4)	0.738
Employed prior to admission		119 (50.2)	49 (47.1)	70 (52.6)	0.441
FCI prior to admission	0	100 (42.2)	43 (41.3)	57 (42.9)	0.812
	1	78 (32.9)	33 (31.7)	45 (33.8)	
	≥2	59 (24.9)	28 (26.9)	31 (23.3)	
Reason for ICU admission	Postoperative care	74 (31.2)	36 (34.6)	38 (28.6)	0.477
	Neurological	66 (27.8)	26 (25.0)	40 (30.1)	
	Cardiovascular	44 (18.6)	16 (15.4)	28 (21.1)	
	Respiratory	38 (16.0)	20 (19.2)	18 (13.5)	
	Trauma	15 (6.3)	6 (5.8)	9 (6.8)	
ICU type	Surgical	104 (43.9)	53 (51.0)	51 (38.3)	0.292
	Cardiovascular	47 (19.8)	18 (17.3)	29 (21.8)	
	Neurological	43 (18.1)	14 (13.5)	29 (21.8)	
	Medical	18 (7.6)	8 (7.7)	10 (7.5)	
	Others	25 (10.5)	11 (10.6)	14 (10.5)	
ICU admission route	ED	110 (46.4)	50 (48.1)	60 (45.1)	0.650
	Others	127 (53.6)	54 (51.9)	73 (54.9)	
Mechanical ventilation use		38 (16.0)	17 (16.3)	21 (15.8)	0.908
Surgery		134 (56.5)	63 (60.6)	71 (53.4)	0.268
Delirium in ICU		25 (10.5)	12 (11.5)	13 (9.8)	0.661
Discharge place	Home	209 (88.2)	93 (89.4)	116 (87.2)	0.602
	LTC	28 (11.8)	11 (10.6)	17 (12.8)	
Severity	APACHE II	10.70 ± 5.81	11.50 ± 6.55	9.97 ± 4.99	0.141
	SAPS	31.92 ± 15.08	34.57 ± 11.91	30.64 ± 16.33	0.259
SOFA		4.37 ± 2.61	4.34 ± 3.10	4.41 ± 1.64	0.908
ICU length of stay, days		4.02 ± 4.28	3.52 ± 4.45	4.41 ± 4.11	0.113
CCI		1.03 ± 1.19	0.95 ± 1.22	1.10 ± 1.17	0.350

Abbreviations: APACHE: acute physiology and chronic health evaluation; CCI: Charlson comorbidity index; ED: emergency department; FCI: functional comorbidity index; ICU: intensive care unit; LTC: long-term care; SAPS: simplified acute physiology score; SOFA: sequential organ failure assessment.

**Table 2:** Changes in scores of PICS variables according to the time after discharge.

Variables	Time			Repeated Measures ANOVA Model			
				Linear		Non-Linear (Quadratic)	
	3 Months	6 Months	12 Months	F	*p*	F	*p*
Anxiety	3.22 ± 3.42	2.76 ± 3.51	3.35 ± 3.64	0.312	0.577	7.242	0.008
Depression	4.70 ± 4.19	3.27 ± 3.57	4.92 ± 4.73	0.646	0.422	53.997	<0.001
PTSD	4.45 ± 5.58	3.98 ± 5.70	5.53 ± 5.83	8.764	0.003	13.238	<0.001
Cognition	25.12 ± 4.15	25.77 ± 3.67	24.06 ± 4.64	11.913	0.001	24.644	<0.001
ADL	7.61 ± 2.09	7.32 ± 1.12	7.39 ± 1.57	3.789	0.053	6.206	0.013

Abbreviations: ADL: activities of daily living; PICS: post-intensive care syndrome; PTSD: post-traumatic stress disorder.

**Table 3:** Differences in the change of PICS scores according to time in groups A and B.

Variable	Group	Time				Group	Time		Group × Time	
		3 Months	6 Months	12 Months			Linear	Non-Linear (Quadratic)	Linear	Non-Linear (Quadratic)
Anxiety	Group A	3.68 ± 3.31	2.44 ± 3.13	3.25 ± 4.00	F-value	0.005	0.091	8.893	4.376	5.465
	Group B	2.85 ± 3.48	3.02 ± 3.77	3.43 ± 3.35	*p*-value	0.942	0.763	0.003	0.038	0.020
Depression	Group A	5.20 ± 3.85	3.28 ± 3.76	3.94 ± 4.50	F-value	0.371	0.058	51.364	23.585	1.085
	Group B	4.30 ± 4.41	3.26 ± 3.43	5.69 ± 4.78	*p*-value	0.543	0.810	0.000	0.000	0.299
PTSD	Group A	5.42 ± 5.97	4.03 ± 6.11	4.71 ± 5.73	F-value	0.036	6.272	13.076	20.338	0.011
	Group B	3.68 ± 5.15	3.95 ± 5.37	6.17 ± 5.85	*p*-value	0.850	0.013	0.000	0.000	0.918
Cognition	Group A	25.75 ± 3.43	25.74 ± 3.89	25.46 ± 4.06	F-value	4.340	8.072	16.019	3.687	11.930
	Group B	24.75 ± 4.49	25.79 ± 3.55	23.24 ± 4.78	*p*-value	0.039	0.005	0.000	0.056	0.001
ADL	Group A	7.82 ± 2.49	7.50 ± 1.51	7.62 ± 2.03	F-value	4.037	3.653	6.350	0.019	0.179
	Group B	7.45 ± 1.69	7.18 ± 0.65	7.22 ± 1.06	*p*-value	0.046	0.057	0.012	0.891	0.673

Abbreviations: ADL: activities of daily living; PICS: post-intensive care syndrome; PTSD: post-traumatic stress disorder.

## Data Availability

Not applicable.

## Supplementary Materials

The following supporting information can be downloaded at: https://www.mdpi.com/article/10.3390/jcm11226653/s1, Table S1: characteristics of all enrolled participants; Table S2: incidence and prevalence of post-intensive care syndrome.
