# Supplementary material for: Post-Intensive Care Syndrome in Non-COVID-19 ICU Survivors during the COVID-19 Pandemic in South Korea: A Multicenter Prospective Cohort Study
Source: J Clin Med. 2022 Nov 9;11(22):6653. doi: 10.3390/jcm11226653 (PMC9699493; doi:10.3390/jcm11226653)
Supplement: Supplementary file 1 [file jcm-11-06653-s001.zip › jcm-2018553-supplementary.pdf]

Supplementary Table S1. Characteristics of All Enrolled Participants

Variables	Categories	Total (N=891)	Followed-up (N=237)	Dropped out (N=654)	p
Age, years		61.09±13.24	58.30±13.30	62.10±13.08	<.001
Male sex		531(59.6)	143(60.3)	338(59.3)	.786
Employed prior to admission		385(43.3)	119(50.4)	266(40.7)	.010
FCI prior to admission	0	364(40.9)	100(42.2)	264(40.4)	.858
	1	305(34.2)	78(32.9)	227(34.7)	
	≥ 2	222(24.9)	59(24.9)	163(24.9)	
Reason for ICU admission	Postoperative care	244(27.4)	74(31.2)	170(26.0)	.166
	Neurological	222(24.9)	66(27.8)	156(23.9)	
	Cardiovascular	179(20.1)	44(18.6)	135(20.6)	
	Respiratory	179(20.1)	38(16.0)	141(21.6)	
	Trauma	67(7.5)	15(6.3)	52(8.0)	
ICU type	Surgical	344(38.6)	104(43.9)	240(36.7)	.055
	Cardiovascular	172(19.3)	47(19.8)	125(19.1)	
	Neurological	164(18.4)	43(18.1)	121(18.5)	
	Medical	114(12.8)	18(7.6)	96(14.7)	
	Others	97(10.9)	25(10.5)	72(11.0)	
ICU Admission route	ED	494(55.4)	110(46.4)	384(58.7)	.001
	Others	397(44.6)	127(53.6)	270(41.3)	
Mechanical ventilation use		178(20.0)	38(16.0)	140(21.4)	.076
Surgery		454(51.0)	134(56.5)	320(49.0)	.047
Delirium in ICU		140(15.7)	25(10.5)	115(17.6)	.011
Discharge place	Home	713(80.0)	209(88.2)	504(77.1)	<.001
	LTC	178(20.0)	28(11.8)	150(22.9)	
Severity	APACHE II	11.79±6.56	10.70±5.81	12.11±6.73	.021
	SAPS	34.48±16.29	31.92±15.08	35.83±16.79	.071
SOFA		4.68±2.79	4.37±2.61	4.79±2.85	.271
ICU length of stay, days		4.81±7.48	4.02±4.28	5.10±8.33	.011
CCI		1.16±1.34	1.03±1.19	1.21±1.38	.065

Abbreviations: APACHE:acute physiology and chronic health evaluation; CCI:charlson comorbidity index; ED:emergency department; FCI:functional comorbidity index; ICU:intensive care unit; LTC:long term care; SAPS:simplified acute physiology score; SOFA:sequential organ failure assessment.

Supplementary Table S2. Incidence and Prevalence of Post-Intensive Care Syndrome

Variables	Time after discharge	Total N=237		Group A N=104		Group B N=133	
		Incidence n(%)	Prevalence n(%)	Incidence n(%)	Prevalence n(%)	Incidence n(%)	Prevalence n(%)
Anxiety	3 mon	26(11.0)	26(11.0)	17(16.3)	17(16.3)	9(6.8)	9(6.8)
	6 mon	14(5.9)	22(9.3)	3(2.9)	6(5.8)	11(8.3)	16(12.0)
	12 mon	13(5.5)	33(13.9)	13(12.5)	17(16.3)	6(4.5)	16(12.0)
Depression	3 mon	54(22.8)	54(22.8)	27(26.0)	27(26.0)	27(20.3)	27(20.3)
	6 mon	9(3.8)	30(12.7)	4(3.8)	13(12.5)	5(3.8)	17(12.8)
	12 mon	37(15.6)	60(25.3)	11(10.6)	20(19.2)	26(19.5)	40(30.1)
PTSD	3 mon	32(13.5)	32(13.5)	20(19.2)	20(19.2)	12(9.0)	12(9.0)
	6 mon	10(4.2)	24(10.1)	5(4.8)	12(11.5)	5(3.8)	12(9.0)
	12 mon	24(10.1)	39(16.5)	11(10.6)	15(14.4)	13(9.8)	24(18.0)
Mental health	3 mon	71(30.0)	71(30.0)	38(36.5)	38(36.5)	33(24.8)	33(24.8)
	6 mon	13(5.5)	43(18.1)	5(4.8)	17(16.3)	8(6.0)	26(19.5)
	12 mon	41(17.3)	73(30.8)	16(15.4)	27(26.0)	25(18.8)	46(34.6)
Cognitive*	3 mon	41(20.9)	41(20.9)	12(16.7)	12(16.7)	29(23.4)	29(23.4)
	6 mon	10(4.2)	31(15.8)	4(3.8)	15(20.8)	6(4.5)	16(12.9)
	12 mon	38(16.0)	58(29.6)	6(5.8)	11(15.3)	32(24.1)	47(37.9)
Physical	3 mon	35(14.8)	35(14.8)	18(17.3)	18(17.3)	17(12.8)	17(12.8)
	6 mon	9(3.8)	30(12.7)	4(3.8)	16(15.4)	5(3.8)	14(10.5)
	12 mon	11(4.6)	26(11.0)	6(5.8)	14(13.5)	5(3.8)	12(9.0)
PICS-overall	3 mon	106(44.7)	106(44.7)	51(49.0)	51(49.0)	55(41.4)	55(41.4)
	6 mon	23(9.7)	91(38.4)	10(9.6)	45(43.3)	13(9.8)	46(34.6)
	12 mon	47(19.8)	112(47.3)	13(12.5)	41(39.4)	34(25.6)	71(53.4)

*N=196 (Group A = 72, Group B =124)

Abbreviations: PICS:post-intensive care syndrome; PTSD:post-traumatic stress disorder.
